# Name MASLD/MASH — and act on it

**DOI:** 10.1097/HC9.0000000000000897

**Published:** 2026-01-09

**Authors:** Jeffrey V. Lazarus, Mário G. Pessoa, Debbie L. Shawcross, Peter Schwarz, Grace L. Su, Simon Barquera

**Affiliations:** 1 City University of New York Graduate School of Public Health and Health Policy, New York, NY, USA; 2 Barcelona Institute for Global Health (ISGlobal), Barcelona, Spain; 3 Faculty of Medicine and Health Sciences, University of Barcelona, Barcelona, Spain; 4 Asociación Latinoamericana para el Estudio del Hígado, Santiago, Chile; 5 Gastroenterology Department, Hospital das Clínicas HCFMUSP, Faculdade de Medicina, Universidade de São Paulo, São Paulo, Brazil; 6 Roger Williams Institute of Liver Studies, King’s College London Faculty of Life Sciences and Medicine, London, UK; 7 Department for Prevention and Care of Diabetes, Medical Faculty Carl Gustav Carusat Theaq, Technical University of Dresden, Dresden, Germany; 8 Paul Langerhans Institute Dresden of the Helmholtz Center Munich at University Hospital and Faculty of Medicine, Dresden, Germany; 9 German Center for Diabetes Research (DZD E.V.), Neuherberg, Germany; 10 Michigan Medicine, Ann Arbor, MI, USA; 11 VA Ann Arbor Healthcare System, Ann Arbor, MI, USA; 12 Nutrition and Health Research Center, National Institute of Public Health, Cuernavaca, Mexico; 13 World Obesity Federation, London, UK

## THE GLOBAL CALL TO ACTION ON MASLD/MASH

The metabolic health crisis is escalating at a dangerous pace around the world, yet one of its most urgent threats – metabolic dysfunction-associated steatotic liver disease (MASLD) and its progressive form, metabolic dysfunction-associated steatohepatitis (MASH)^1^ – remains largely invisible in the global health agenda. This silence is not only unacceptable, it is unsustainable.

MASLD and MASH are not niche conditions; they are diseases in their own right, with over a quarter of the world’s population living with them, which act as accelerants of cancer, cardiovascular disease, and diabetes in a complex interplay of metabolic conditions. But, critically, they are not two separate diseases; MASLD and MASH exist on a spectrum – just as HIV and AIDS do. Ignoring the early stage of this dynamic spectrum (MASLD), while only acknowledging its advanced stage (MASH), is not only clinically misguided but also a profound failure of prevention.

If the world is serious about reducing premature death and disability from non-communicable diseases (NCDs), then the World Health Organization (WHO) must formally recognise MASLD/MASH as a priority NCD spectrum, alongside other high prevalence diseases, and include it as such in its agenda.^2^

**FIGURE 1:**
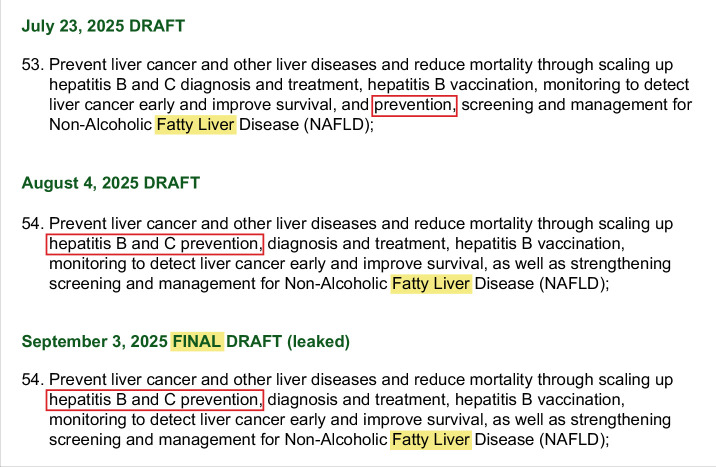
The evolution of NAFLD’s inclusion in the draft political declaration of the fourth United Nations General Assembly High-Level Meeting on non-communicable diseases.

The fourth United Nations (UN) General Assembly High-Level Meeting on NCDs, held on 25th September 2025 in New York City, marked a major step forward in advancing said recognition and inclusion. However, it was also concerning, as the outdated and stigmatising name non-alcoholic fatty liver disease (NAFLD)^3^ was used (Figure 1) and MASLD/MASH was still not recognised as a priority NCD on par with other highly prevalent diseases. This move sends the wrong signal to people living with MASLD/MASH. Thus, a liver-focused metabolic health side-event,^4^ led by the Healthy Livers Healthy Lives Coalition and the Barcelona Institute for Global Health (ISGlobal) Public Health Liver Group, called for decisive global policy to rectify this shortcoming (Box 1).

**BOX 1:**
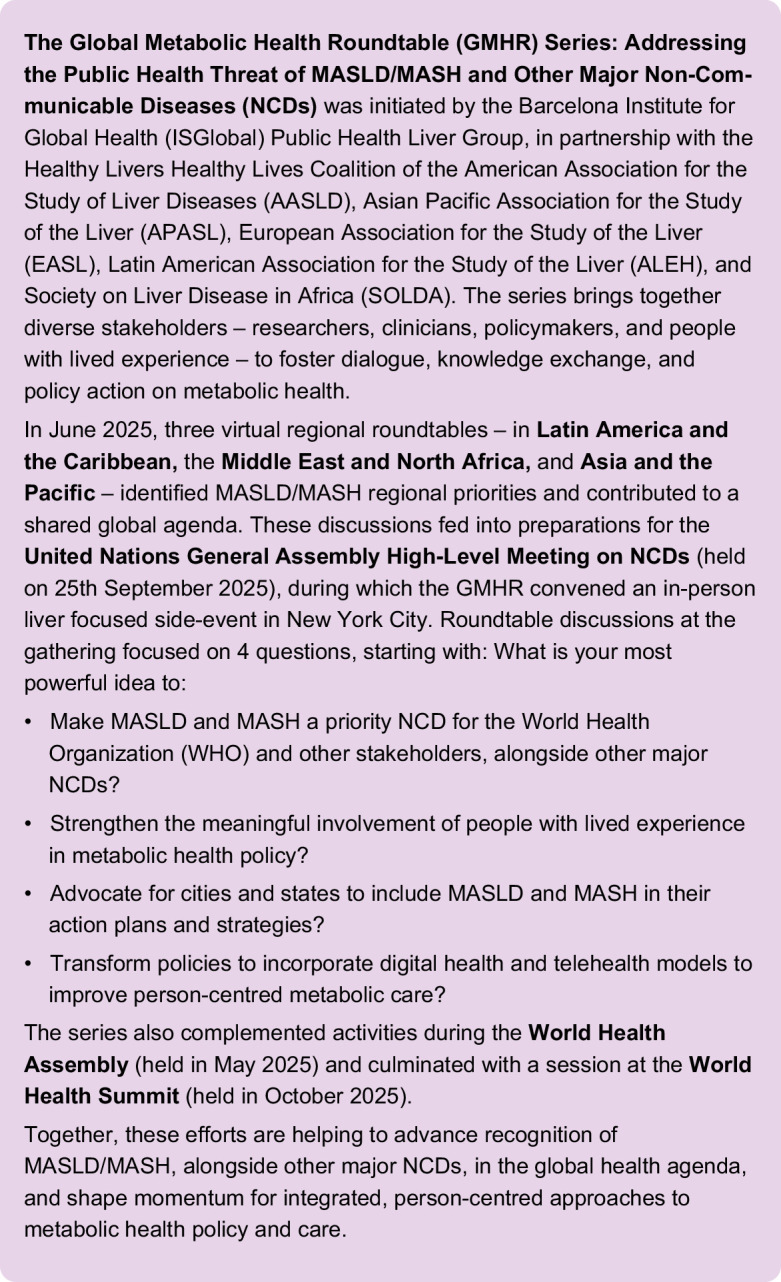
The Global Metabolic Health Roundtable Series: Addressing the Public Health Threat of MASLD/MASH and Other Major Non-Communicable Diseases.

WHO sets the tone and direction for global health policy through normative guidance, action plans, and strategies. Without clear disease recognition and guidance from WHO, the mandate, metrics, and momentum needed to act decisively will remain elusive. The result? Hundreds of millions of people worldwide remain undiagnosed, unsupported, and at risk of preventable disease progression.

## ELEVATING THE VOICES THAT MATTER MOST

One of the clearest gaps in liver and metabolic health policy is the lack of representation of people living with MASLD/MASH. They are too often underdiagnosed, underprioritised, and left out of conversations that shape their care. Progress begins with listening.^5^

WHO should actively support frameworks for the meaningful involvement of people with lived experience in policy and programme design. Their testimonies cut through statistics, highlighting barriers to care, stigma in health systems, and the human toll of a late diagnosis. Cross-collaboration among patient advocates, NCD organisations, medical associations, and researchers can unify voices and create a movement too strong to ignore. The GMHR Series embodies this and was endorsed by the City University of New York Graduate School of Public Health and Health Policy (CUNY SPH), the Fatty Liver Alliance, the Global Think-tank on Steatotic Liver Disease, the International Diabetes Federation (IDF), the International Liver Cancer Movement (ILCM), the MASH Cities Coalition, and UNITE, the Parliamentarians Network for Global Health.

Advocates – from liver health, diabetes, and obesity organisations to broader NCD coalitions – now stand ready to champion this MASLD/MASH cause. Clinical practice guidelines^6^^–^^11^ and patient guidance^12^^,^^13^ for MASLD/MASH exist, but without proper recognition by the UN and WHO, efforts remain fragmented and underpowered. WHO’s inclusion of MASLD/MASH as a unified disease spectrum would provide the legitimacy, accountability, and global signal needed to unlock investment, mobilise governments, and accelerate action at every level.

To correct the MASLD/MASH course, a blueprint for WHO action should include:

- Recognise MASLD and MASH as a single dynamic disease spectrum, so that prevention and treatment strategies cover the full continuum.
- Embed MASLD/MASH explicitly into NCD normative guidance and action plans.
- Establish epidemiological targets and indicators to monitor MASLD/MASH progress, as is already done for other major NCDs.
- Name MASLD/MASH in the implementation of WHO “best buys”, including in fiscal policies, food environment reforms, the regulation of marketing of unhealthy products, front-of-pack labelling on ultraprocessed foods and sugar-sweetened beverages, and health-promoting taxation.
- Integrate MASLD/MASH into digital health, primary care, and screening strategies, as part of person-centred and equitable care across all health systems.
- Institutionalise engagement so that lived experience shapes policy from the start, instead of being an afterthought.

Our call is simple but urgent: the UN and WHO must name MASLD and MASH for what they are – a dynamic continuum of metabolic liver disease – and place them at the centre of the global NCD agenda, alongside other major NCDs.^14^^,^^15^ Anything less risks condemning hundreds of millions of people to preventable disease and undermines the global pledge to reduce premature NCD deaths by 2030. The world cannot wait. The time for WHO to act is now.

## References

[1] Huang DQ Wong VWS Rinella ME Boursier J Lazarus JV Yki-Järvinen H. Metabolic dysfunction-associated steatotic liver disease in adults. Nat Rev Dis Primers. 2025;11:14. 40050362 10.1038/s41572-025-00599-1

[2] Lazarus JV Agirre-Garrido L White TM. Best buys for metabolic dysfunction-associated steatotic liver disease and metabolic dysfunction-associated steatohepatitis: a global Delphi study. Lancet Gastroenterol Hepatol 2025. 10.1016/S2468-1253(25)00300-0 41397405

[3] Rinella ME Lazarus JV Ratziu V Francque SM Sanyal AJ Kanwal F. A multisociety Delphi consensus statement on new fatty liver disease nomenclature. Hepatology. 2023;78:1966–1986. 37363821 10.1097/HEP.0000000000000520 PMC10653297

[4] Global Metabolic Health. MASLD/MASH and other major NCDS. Devex; 2025. Accessed 7 October 2025. https://pages.devex.com/global-metabolic-health-roundtable.html

[5] Lazarus JV Ivancovsky Wajcman D Pannain S Brennan PN Manolas MI Jepsen P. The people-first liver charter. Nat Med. 2025;31:2109–2116. 40473951 10.1038/s41591-025-03759-8

[6] Rinella ME Neuschwander-Tetri BA Siddiqui MS Abdelmalek MF Caldwell S Barb D. AASLD Practice Guidance on the clinical assessment and management of nonalcoholic fatty liver disease. Hepatology. 2023;77:1797–1835. 36727674 10.1097/HEP.0000000000000323 PMC10735173

[7] Cusi K Abdelmalek MF Apovian CM Balapattabi K Bannuru RR Barb D. Metabolic dysfunction–associated steatotic liver disease (MASLD) in people with diabetes: the need for screening and early intervention. A consensus report of the American diabetes association. Diabetes Care. 2025;48:1057–1082. 40434108 10.2337/dci24-0094

[8] Cusi K Isaacs S Barb D Basu R Caprio S Garvey WT. American association of clinical endocrinology clinical practice guideline for the diagnosis and management of nonalcoholic fatty liver disease in primary care and endocrinology clinical settings: Co-sponsored by the American association for the study of liver diseases (AASLD). Endocr Pract. 2022;28:528–562. 35569886 10.1016/j.eprac.2022.03.010

[9] Tacke F Horn P Wai-Sun Wong V Ratziu V Bugianesi E Francque S. EASL–EASD–EASO Clinical Practice Guidelines on the management of metabolic dysfunction-associated steatotic liver disease (MASLD). J Hepatol. 2024;81:492–542. 38851997 10.1016/j.jhep.2024.04.031

[10] Diaz LA Arab JP Idalsoaga F Perelli J Vega J Dirchwolf M. Updated recommendations for the management of metabolic dysfunction–associated steatotic liver disease (MASLD) by the Latin American working group. Ann Hepatol. 2025;30:101903101903. 10.1016/j.aohep.2025.101903 40089151

[11] Eslam M Fan J-G Yu M-L Wong VWS Cua IH Liu CJ. The Asian Pacific association for the study of the liver clinical practice guidelines for the diagnosis and management of metabolic dysfunction-associated fatty liver disease. Hepatol Int. 2025;19:261–301. 40016576 10.1007/s12072-024-10774-3

[12] American Association of Clinical Endocrinology. AACE Patient Guide to Metabolic Dysfunction–Associated Steatotic Liver Disease (MASLD) and Metabolic Dysfunction–Associated Steatohepatitis (MASH). Accessed October 7, 2025. https://www.aace.com/disease-and-conditions/masldmash/aace-patient-guide-masld-mash

[13] Francque SM Marchesini G Kautz A Walmsley M Dorner R Lazarus JV. Non-alcoholic fatty liver disease: a patient guideline. JHEP Rep. 2021;3:100322100322. 10.1016/j.jhepr.2021.100322 PMC8514420 34693236

[14] Lazarus JV Schwarz P Barquera S. Opinion: The liver — a metabolic health blindspot on the global NCD agenda. Devex; 2025. Accessed October 1, 2025. https://www.devex.com/news/sponsored/opinion-the-liver-a-metabolic-health-blindspot-on-the-global-ncd-agenda-110799

[15] Lazarus JV Mark HE White TM. A call to action from the Global Think-tank on Steatotic Liver Disease. Nature Health 2026. 10.1038/s44360-025-00042-5

